# Emergency Department Management of Suspected Calf-Vein Deep Venous Thrombosis: A Diagnostic Algorithm

**DOI:** 10.5811/westjem.2016.5.29951

**Published:** 2016-06-28

**Authors:** Levi Kitchen, Matthew Lawrence, Matthew Speicher, Kenneth Frumkin

**Affiliations:** Naval Medical Center Portsmouth, Emergency Department, Portsmouth, Virginia

## Abstract

**Introduction:**

Unilateral leg swelling with suspicion of deep venous thrombosis (DVT) is a common emergency department (ED) presentation. Proximal DVT (thrombus in the popliteal or femoral veins) can usually be diagnosed and treated at the initial ED encounter. When proximal DVT has been ruled out, isolated calf-vein deep venous thrombosis (IC-DVT) often remains a consideration. The current standard for the diagnosis of IC-DVT is whole-leg vascular duplex ultrasonography (WLUS), a test that is unavailable in many hospitals outside normal business hours. When WLUS is not available from the ED, recommendations for managing suspected IC-DVT vary. The objectives of the study is to use current evidence and recommendations to (1) propose a diagnostic algorithm for IC-DVT when definitive testing (WLUS) is unavailable; and (2) summarize the controversy surrounding IC-DVT treatment.

**Discussion:**

The Figure combines D-dimer testing with serial CUS or a single deferred FLUS for the diagnosis of IC-DVT. Such an algorithm has the potential to safely direct the management of suspected IC-DVT when definitive testing is unavailable. Whether or not to treat diagnosed IC-DVT remains widely debated and awaiting further evidence.

**Conclusion:**

When IC-DVT is not ruled out in the ED, the suggested algorithm, although not prospectively validated by a controlled study, offers an approach to diagnosis that is consistent with current data and recommendations. When IC-DVT is diagnosed, current references suggest that a decision between anticoagulation and continued follow-up outpatient testing can be based on shared decision-making. The risks of proximal progression and life-threatening embolization should be balanced against the generally more benign natural history of such thrombi, and an individual patient’s risk factors for both thrombus propagation and complications of anticoagulation.

## INTRODUCTION

### Clinical Scenario

Our interest in this topic was prompted by two emergency department (ED) visits by an 84-year-old man. Initially, he presented with right calf swelling 10 days after shoulder surgery. Bedside compression ultrasound (CUS) was negative for proximal deep venous thrombosis (DVT), and a D-dimer was elevated at 3.3μg/mL. Right calf DVT was strongly suspected and he was treated with therapeutic enoxaparin. Whole leg ultrasound (WLUS) 36 hours later diagnosed chronic DVT in the right gastrocnemius veins. Therapeutic enoxaparin was continued by his physicians. He returned to our ED six days after his initial visit with right shoulder pain and an 18cmx7cm chest wall hematoma with evidence of active bleeding. Inpatient management consisted of protamine reversal of his enoxaparin and transfusion of blood and platelets.

### Background

In emergency patients, acute unilateral leg pain and/or swelling are common complaints, often prompting a search for DVT. Proximal DVT (with its risk for pulmonary embolism [PE]) is commonly ruled in or out during the initial ED encounter.^1^,^2^ As in our patient, when proximal DVT is eliminated, isolated calf deep vein thrombosis (IC-DVT) often remains in the differential diagnosis. Whole-leg duplex ultrasonography (WLUS), the current standard for an affirmative diagnosis of IC-DVT, is unavailable after-hours in many EDs.^3^,^4^ The purpose of this article is to suggest an algorithm for the evaluation of patients with suspected IC-DVT when WLUS is unavailable. Treatment controversies surrounding this entity are described.

## DISCUSSION

### The Nature of the Problem

No one would deny the frequency and importance of DVT, which affects around one in 1,000 persons per year.^5^ Emergency physicians appropriately have a high degree of concern for this condition. We look for it frequently, and DVT is found in 10–25% of patients in whom it is suspected.^6^ We seek to diagnose proximal DVT to prevent PE and the postthrombotic syndrome. When proximal DVT is ruled out, distal thrombus must often still be considered. We pursue the diagnosis of IC-DVT out of concern for the progression of these distal thrombi to proximal DVT and PE. In community practice, isolated calf DVT was diagnosed in 11% of 1,495 patients in whom it was suspected.^7^ When all patients undergo WLUS, IC-DVT is even more frequently found, representing about 50% of diagnosed DVTs.^8^ The majority of distal thrombi are non-obstructive and asymptomatic and long-term outcomes are similar in patients diagnosed using either proximal or whole-leg imaging.^9^,^10^

### IC-DVT: Risk of Thrombus Propagation, Mortality, and Pulmonary Embolism

All DVT is assumed to start in the calf veins.^11^ Untreated, symptomatic IC-DVT progresses to involve the popliteal or femoral veins ≤16% of the time.^1^,^12^–^14^ Such propagation has not been documented after two weeks.^13^,^15^–^17^ Risk factors promoting propagation include a history of cancer, inpatient status, positive D-dimer, extensive thrombus or proximity to proximal veins, absence of reversible provoking factors for DVT, history of trauma and history of prior venous thromboembolism (VTE).^13^

Calf vein DVT, with or without treatment, has a mortality of ≤1%.^1^ When the search for DVT begins after a diagnosis of PE is made, 7–11% of patients with suspected symptomatic PE will have IC-DVT.^18^ If tested, 13% of patients with proven IC-DVT will have evidence of “silent” PE.^19^ The controversy surrounding the significance of diagnosing and treating small or minimally symptomatic PEs is under active discussion, and is not covered here.^20^,^21^

### How Should the Diagnosis of Suspected IC-DVT Be Approached?

When available, WLUS rules out IC-DVT with a subsequent composite VTE complication rate of ≤1%.^5^,^8^,^10^,^22^–^24^ In the absence of WLUS, commonly available diagnostic modalities are bedside (or radiology department) proximal compression ultrasonography, clinical probability assessments, and D-dimer testing.

### The Role of D-dimer Testing, Pretest Clinical Probability, and Compression Ultrasonography

D-dimer and Clinical Probability: For both DVT in general, and isolated calf DVT specifically, the negative predictive value (NPV) of a D-dimer in low-risk patients (Wells score of zero or less) is≥99%.^6^,^25^ The 2012 American College of Chest Physicians (ACCP) Clinical Practice Guidelines for Antithrombotic Therapy and Prevention of Thrombosis endorses a strategy for diagnosing DVT combining D-dimer testing with pretest probability assessment using the Wells score.^5^ In patients with a negative D-dimer and a low pretest probability of first lower extremity DVT, Wells et al. 2003 and 2006 and the ACCP 2012 guidelines support no further testing.^5^,^6^,^26^ With an elevated D-dimer, ACCP recommendations are for proximal compression ultrasound.

Compression Ultrasound (CUS): In the absence of WLUS, the presence of a positive D-dimer or a moderate or high clinical probability Wells score should be followed by compression ultrasonography in the ED to rule out proximal DVT. A positive CUS would identify the need for therapeutic anticoagulation. The significant numbers of emergency physicians trained in bedside CUS make that modality increasingly more accessible and often more available than radiology studies, particularly outside normal business hours. Multiple studies have demonstrated that proximal DVT can reliably be diagnosed or excluded in the ED with bedside proximal CUS with sensitivities of 95–99%.^27^–^30^ Formal radiology CUS remains an option when available. The additional value of initially combining both CUS and a D-dimer has yet to be specifically studied. However, when both tests are done and negative, the combination effectively excludes any clinically significant DVT (≥99% NPV).^10^,^26^,^31^–^34^ The combination has been recommended in patients with high clinical pretest probability.^5^

## RECOMMENDATIONS

### Diagnosis of IC-DVT in the Setting of Positive D-dimer and Negative CUS for Proximal DVT

When the D-dimer is positive and CUS is negative, WLUS is the definitive diagnostic test and the procedure of choice. When WLUS is not immediately available, the ACCP recommends two strategies presented in the Figure: either direct imaging of the calf veins with a short-term definitive whole-leg ultrasound, or a repeat proximal CUS in a week to assess for proximal progression.^5^ The 1-week repeat CUS has been found to be both equivalent to a single WLUS in ruling out IC-DVT likely to progress, and safe (0–1.8% VTE at 3–6 months).^10^,^23^,^31^–^36^

For the many emergency patients for whom outpatient testing and follow up cannot be reliably arranged, the ability to rule out proximal propagation of suspected IC-DVT with repeat ED bedside compression ultrasound, makes return to the ED for such testing an option.

### Bridging Anticoagulation

When proximal DVT has been ruled out in the ED and suspected IC-DVT is being investigated with planned short-term deferred WLUS or repeat proximal CUS, the practice of providing a bridge of empiric anticoagulation between imaging studies is not supported.^4^,^10^,^23^,^31^–^34^,^36^

### Treatment of Confirmed IC-DVT - Selective Anticoagulation is Controversial

We present an algorithm for the diagnosis of IC-DVT when definitive WLUS is not immediately available. Treatment for IC-DVT is controversial, and will only be briefly reviewed here.^1^,^9^,^14^,^36^–^40^ Previous ACCP guidelines, current European guidelines and commonly used references (UpToDate) recommend treating IC-DVT with at least three months of anticoagulation.^41^,^42^,^43^ The latest ACCP guidelines include a more selective approach.^13^ The controversy is best exemplified by a survey of faculty physicians at a major U.S, medical center. Half of respondents would “routinely use anticoagulation to treat venous thrombosis below the knee” and half would not.^44^ There is a near-universal call for large randomized trials to address the question. One such trial is underway (www.ClinicalTrials.gov).^45^ In the absence of new and definitive data, and as suggested by the ACCP, recommendations to base treatment decisions on risk/benefit analysis and shared decision-making are becoming more common.^1^,^12^,^13^

The controversy over treatment largely derives from an increase in the frequency of diagnosis of IC-DVT, coupled with conclusions that distal DVT is less concerning than proximal. When WLUS is used instead of CUS, the reported prevalence of distal DVT rises to half of all lower extremity DVTs.^36^ However, risk factors associated with distal DVTs are more commonly transient and reversible, and mortality and recurrence rates are less.^18^,^46^,^47^ Those in favor of observation rather than treatment for IC-DVT note that untreated patients with negative proximal CUS (many of whom would likely have IC-DVT if looked for) demonstrate an acceptable outcome profile without treatment.^14^,^36^ Treating them all exposes patients to unnecessary bleeding complications.^18^,^23^,^36^,^48^ Our patient is an example.

### Selective Treatment of Confirmed IC-DVT - Shared Decision-Making

The ACCP evidence-based clinical practice guidelines (currently in their 10th edition, spanning 30 years) provide a solid starting point for clinical decision-making.^5^,^13^,^49^,^50^ The most recent edition offers two options for confirmed IC-DVT: (1) therapeutic anticoagulation or (2) weekly surveillance with compression ultrasonography for two weeks to monitor for proximal thrombus propagation.^13^ They suggest that those with severe symptoms or with risk factors for proximal extension should receive anticoagulation. Patients at risk for anticoagulation-associated major bleeding (see Table 11, Kearon et al., 2016) may be better served by surveillance. For those at lower risk for both propagation and hemorrhage there may be room to consider a more selective approach using shared decision-making.^13^,^14^,^51^ Discussions should be well documented and focus on the patient’s valuation of, and ability to comply with, serial surveillance for clot propagation versus their tolerance for the risks of bleeding associated with prevention. Given the controversy over IC-DVT treatment, the patient’s primary provider and/or consultants should be involved in the decision-making whenever possible, with every effort to assure close follow up. There is a lack of data comparing management strategies for IC-DVT in patients with varying levels of these conflicting risks.

### Therapeutic Adjuncts

The role of compression stockings for comfort and for the prevention postthrombotic syndrome (PTS) has not been studied for IC-DVT. For proximal DVT, adverse events from stockings are rare and minor, but their value for preventing PTS is “in doubt.”^52^–^54^ No recommendations could be found for the role of aspirin in the treatment of IC-DVT.

## LIMITATIONS

Data on the prevalence of DVT overall and the subset of IC-DVT vary significantly. While the number of reports is considerable, many are derived from small underpowered observational cohort studies, subsequently folded into meta-analyses. Explanations for variability include the size and heterogeneity of the patient population (inpatient, outpatient, community, post-surgical, trauma, presence or absence of symptoms), the reason for testing (suspected or confirmed PE, versus DVT), and the diagnostic imaging used. Most series did not image the entire leg.

The algorithm suggested is based on the latest evidence and practice guidelines. Like so much of the literature on this topic, it would benefit from prospective controlled evaluation.

Any strategy involving compliance with return visits (surveillance) loses some patients to follow up.^18^,^55^ During the period covered by this discussion, D-dimer assays evolved and the Wells clinical prediction rules were modified.^6^,^26^,^56^ Current recommendations are predicated on the use of high-sensitivity D-dimer assays.^5^,^57^ Multiple such assays are in use.^58^ Both the Wells criteria and D-dimer assays have greater sensitivity for proximal than isolated distal DVT.^25^,^59^–^61^

Leg pain and swelling are among the common ED complaints that trigger a search for serious conditions requiring urgent intervention. Yet<25% will have DVT. Even applying clinical decision rules and diagnostic tests with 99% sensitivity, physicians will see false negatives with serious consequences, as seen in multiple case reports available in the literature.^62^–^64^ Clinical judgment, “high index of suspicion,” patient education, comprehensive discharge instructions, and close follow up remain tools we need to routinely apply.

Muscular calf vein thrombosis: Roughly half of calf vein thromboses are isolated to the veins of the soleus and gastrocnemius muscles.^65^ Although these are most often considered “deep” veins, thrombosis confined to the muscular veins has a “lower risk of extension than thrombosis that involves the axial (i.e., true deep; peroneal, tibial) veins.”^13^ Although subject to similar variability in opinion as DVT treatment in general, anticoagulation of calf muscle thrombosis is less commonly favored.^15^,^66^,^67^

## CONCLUSION

Unilateral leg pain/swelling is a common ED complaint. The diagnosis of isolated calf vein DVT is particularly challenging when the definitive diagnostic study, whole-leg ultrasound, is unavailable. An ED diagnostic algorithm is presented for this situation, based on the most recent recommendations of the American College of Chest Physicians. It is important to remember that this algorithm is based on critical appraisal of the current literature and will require prospectively controlled studies before it can be recommended for widespread implementation. Treatment is controversial: universal versus selective anticoagulation. The risks of proximal progression and life-threatening embolization should be considered along with the generally more benign natural history of distal clots and an individual patient’s risk factors for both clot propagation and the complications of therapy.

## Figures and Tables

**Figure:**
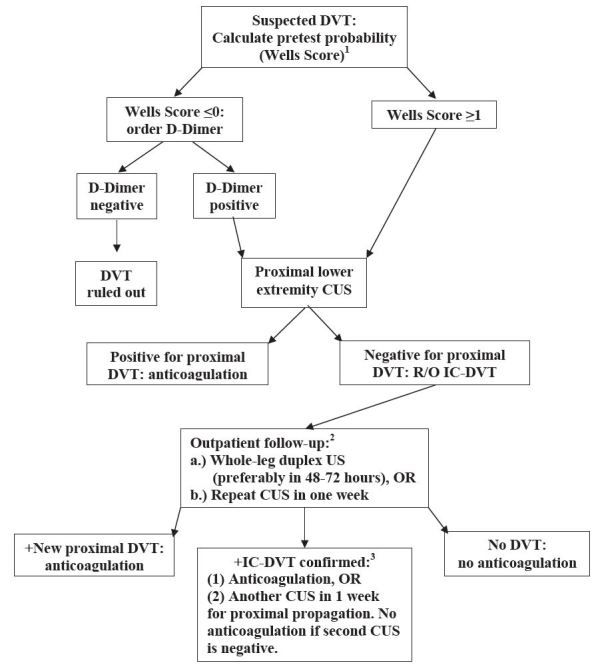
Proposed emergency department DVT evaluation algorithm when full-leg vascular duplex ultrasonography is unavailable. *ACCP,* American College of Chest Physicians; *CUS,* compression ultrasound; *DVT,* deep venous thrombosis; *IC-DVT,* isolated calf deep venous thrombosis; *R/O,* rule out; *US,* ultrasound. 1. The pretest probability of DVT is most frequently assessed with the clinical model developed by Wells, et al.[6] One point is added for each of the following positive findings: (i) active cancer (treatment ongoing or within the previous 6 months, or palliative); (ii) paralysis, paresis or recent plaster immobilization of the lower extremities; (iii) recently bedridden for 3 days or more, or major surgery within the previous 12 weeks requiring general or regional anesthesia; (iv) localized tenderness along the distribution of the deep venous system; (v) entire leg swelling; (vi) calf swelling at least 3 cm larger than that on the asymptomatic leg (measured 10 cm below the tibial tuberosity); (vii) pitting edema confined to the symptomatic leg; (viii) collateral superficial veins (nonvaricose); and (ix) previously documented DVT. Two points are subtracted from the total if an alternative diagnosis is at least as likely as DVT. Based on this checklist the clinical probability of DVT is assessed as low if the score is ≤0, moderate (a score of 1 or 2), or high (a score of ≥ 3). The ability of a negative D-dimer to rule out DVT at a given pretest clinical probability (Well’s score) is dependent upon the sensitivity of the specific assay used. When a negative high-sensitivity D-dimer is combined with a low (≤0) or moderate (≤2) Well’s score, the negative predictive value for DVT is 99%. This is reflected in the algorithm. Wells, et al. (2006) conclude that with moderate sensitivity D-dimer tests “the negative LRs are not sufficiently low to exclude DVT without ultrasound among patients with moderate and high pretest probability estimates” (Well’s score ≥ 1). [6] 2. The practice of providing a bridge of empiric anticoagulation between imaging studies is not supported.[10, 23, 31–34, 36] 3. Per ACCP and others, the decision to anti-coagulate confirmed IC-DVT (versus conservative therapy) benefits from a thorough risk/benefit analysis and shared decision-making. Risk factors for extension of confirmed IC-DVT include positive D-dimer, severe symptoms, thrombosis that is extensive or close to the proximal veins, absence of reversible provoking factors for DVT, active cancer, a history of venus thromboembolism (VTE), and inpatient status. Those at higher risk for bleeding complications from anticoagulation may be better served by continued surveillance with compression ultrasonography alone (Kearon, et al.; Table 11).[13,14] The patient’s primary provider and/or consultants should be involved in the decision-making whenever possible, with every effort to assure close follow up.

## References

[1] Horner D, Hogg K, Body R (2016). Should we be looking for and treating isolated calf vein thrombosis?. Emerg Med J.

[2] Horner D (2012). Isolated distal deep vein thrombosis in symptomatic ambulatory patients: a prospective data analysis and therapeutic feasibility study. https://www.escholar.manchester.ac.uk/uk-ac-man-scw:186165.

[3] Aboulafia ED, Lawrence L, DeLong R (2001). Can an algorithm predict appropriate utilization of emergency venous studies?. Int J Angiol.

[4] Arnaoutakis GJ, Pirrucello J, Brooke BS (2010). Venous duplex scanning for suspected deep vein thrombosis: results before and after elimination of after-hours studies. Vasc Endovascular Surg.

[5] Bates SM, Jaeschke R, Stevens SM (2012). Diagnosis of DVT: antithrombotic therapy and prevention of thrombosis, 9^th^ ed. American College of Chest Physicians Evidence-Based Clinical Practice Guidelines. Chest.

[6] Wells PS, Owen C, Doucette S (2006). Does this patient have deep vein thrombosis?. JAMA.

[7] Spencer FA, Kroll A, Lessard D (2012). Isolated calf deep vein thrombosis in the community setting: the Worcester Venous Thromboembolism study. J Thromb Thrombolysis.

[8] Johnson SA, Stevens SM, Woller SC (2010). Risk of deep vein thrombosis following a single negative whole-leg compression ultrasound: a systematic review and meta-analysis. JAMA.

[9] Cohen AT, SB, Fassiadis N (2008). Are isolated distal deep-vein thromboses clinically significant?. Therapy.

[10] Bernardi E, Camporese G, Buller HR (2008). Serial 2-point ultrasonography plus D-dimer vs whole-leg color-coded Doppler ultrasonography for diagnosing suspected symptomatic deep vein thrombosis: a randomized controlled trial. JAMA.

[11] Kearon C (2003). Natural history of venous thromboembolism. Circulation.

[12] De Martino RR, Wallaert JB, Rossi AP (2012). A meta-analysis of anticoagulation for calf deep venous thrombosis. J Vasc Surg.

[13] Kearon C, Akl EA, Ornelas J (2016). Antithrombotic therapy for VTE disease: CHEST guideline and expert panel report. Chest.

[14] Masuda EM, Kistner RL, Musikasinthorn C (2012). The controversy of managing calf vein thrombosis. J Vasc Surg.

[15] Macdonald PS, Kahn SR, Miller N (2003). Short-term natural history of isolated gastrocnemius and soleal vein thrombosis. J Vasc Surg.

[16] Masuda EM, Kessler DM, Kistner RL (1998). The natural history of calf vein thrombosis: lysis of thrombi and development of reflux. J Vasc Surg.

[17] Solis MM, Ranval TJ, Nix ML (1992). Is anticoagulation indicated for asymptomatic postoperative calf vein thrombosis?. J Vasc Surg.

[18] Palareti G, Schellong S (2012). Isolated distal deep vein thrombosis: what we know and what we are doing. J Thromb Haemost.

[19] Stein PD, Matta F, Musani MH (2010). Silent pulmonary embolism in patients with deep venous thrombosis: a systematic review. Am J Med.

[20] Morgan C, Choi H (2015). Towards evidence-based emergency medicine:best BETs from the Manchester Royal Infirmary. BET 1: Do patients with a clinically suspected subsegmental pulmonary embolism need anticoagulation therapy?. Emerg Med J.

[21] Newman DH, Schriger DL (2011). Rethinking testing for pulmonary embolism: less is more. Ann Emerg Med.

[22] Gibson NS, Schellong SM, Kheir DY (2009). Safety and sensitivity of two ultrasound strategies in patients with clinically suspected deep venous thrombosis: a prospective management study. J Thromb Haemost.

[23] Guanella R, Righini M (2012). Serial limited versus single complete compression ultrasonography for the diagnosis of lower extremity deep vein thrombosis. Semin Respir Crit Care Med.

[24] Horner D, Hogg K, Body R (2014). Single whole-leg compression ultrasound for exclusion of deep vein thrombosis in symptomatic ambulatory patients: a prospective observational cohort study. Br J Haematol.

[25] Sartori M, Cosmi B, Legnani C (2012). The Wells rule and D-dimer for the diagnosis of isolated distal deep vein thrombosis. J Thromb Haemost.

[26] Wells PS, Anderson DR, Rodger M (2003). Evaluation of D-dimer in the diagnosis of suspected deep-vein thrombosis. N Engl J Med.

[27] Burnside PR, Brown MD, Kline JA (2008). Systematic review of emergency physician-performed ultrasonography for lower-extremity deep vein thrombosis. Acad Emerg Med.

[28] Crisp JG, Lovato LM, Jang TB (2010). Compression ultrasonography of the lower extremity with portable vascular ultrasonography can accurately detect deep venous thrombosis in the emergency department. Ann Emerg Med.

[29] Lewiss RE, Kaban NL, Saul T (2013). Point-of-care ultrasound for a deep venous thrombosis. Glob Heart.

[30] Pomero F, Dentali F, Borretta V (2013). Accuracy of emergency physician-performed ultrasonography in the diagnosis of deep-vein thrombosis: a systematic review and meta-analysis. Thromb Haemost.

[31] Bernardi E, Prandoni P, Lensing AW (1998). D-dimer testing as an adjunct to ultrasonography in patients with clinically suspected deep vein thrombosis: prospective cohort study. The Multicentre Italian D-dimer Ultrasound Study Investigators Group. BMJ.

[32] Kearon C, Ginsberg JS, Douketis J (2005). A randomized trial of diagnostic strategies after normal proximal vein ultrasonography for suspected deep venous thrombosis: D-dimer testing compared with repeated ultrasonography. Ann Intern Med.

[33] Kraaijenhagen RA, Piovella F, Bernardi E (2002). Simplification of the diagnostic management of suspected deep vein thrombosis. Arch Intern Med.

[34] Tick LW, Ton E, van Voorthuizen T (2002). Practical diagnostic management of patients with clinically suspected deep vein thrombosis by clinical probability test, compression ultrasonography, and D-dimer test. Am J Med.

[35] Cogo A, Lensing AW, Koopman MM (1998). Compression ultrasonography for diagnostic management of patients with clinically suspected deep vein thrombosis: prospective cohort study. BMJ.

[36] Righini M, Paris S, Le Gal G (2006). Clinical relevance of distal deep vein thrombosis. Review of literature data. Thromb Haemost.

[37] Galanaud JP, Sevestre MA, Genty C (2014). Incidence and predictors of venous thromboembolism recurrence after a first isolated distal deep vein thrombosis. J Thromb Haemost.

[38] Guarnera G, Abeni D, Antignani PL (2014). Update on distal deep venous thrombosis. Reports of a multicenter study. Int Angiol.

[39] Masuda EM, Kistner RL (2010). The case for managing calf vein thrombi with duplex surveillance and selective anticoagulation. Dis Mon.

[40] Palareti G (2014). How I treat isolated distal deep vein thrombosis (IDDVT). Blood.

[41] Kearon C, Kahn SR, Agnelli G (2008). Antithrombotic therapy for venous thromboembolic disease: American College of Chest Physicians Evidence-Based Clinical Practice Guidelines (8^th^ Edition). Chest.

[42] Nicolaides AN, Fareed J, Kakkar AK (2013). Prevention and treatment of venous thromboembolism—International Consensus Statement. Int Angiol.

[43] Bauer KA, Leung LAK, Mandel J Approach to the diagnosis and therapy of lower extremity deep vein thrombosis. UpToDate.

[44] Anstadt MJ, Robertson TC, Milner R (2014). No consensus exists for use of anticoagulation for calf vein thrombosis. Vascular.

[45] Righini M, Kahn S (2000). Randomized controlled trial of anticoagulation vs placebo for a first symptomatic isolated distal deep-vein thrombosis (IDDVT) (CACTUS-PTS). ClinicalTrialsgov [Internet].

[46] Galanaud JP, Genty C, Sevestre MA (2011). Predictive factors for concurrent deep-vein thrombosis and symptomatic venous thromboembolic recurrence in case of superficial venous thrombosis. The OPTIMEV study. Thromb Haemost.

[47] Galanaud JP, Quenet S, Rivron-Guillot K (2009). Comparison of the clinical history of symptomatic isolated distal deep-vein thrombosis vs. proximal deep vein thrombosis in 11,086 patients. J Thromb Haemost.

[48] Righini M (2007). Is it worth diagnosing and treating distal deep vein thrombosis? No. J Thromb Haemost.

[49] Guyatt GH, Akl EA, Crowther M (2012). Executive summary: Antithrombotic Therapy and Prevention of Thrombosis, 9^th^ ed: American College of Chest Physicians Evidence-Based Clinical Practice Guidelines. Chest.

[50] Guyatt GH, Akl EA, Crowther M (2012). Introduction to the ninth edition: Antithrombotic Therapy and Prevention of Thrombosis, 9^th^ ed: American College of Chest Physicians Evidence-Based Clinical Practice Guidelines. Chest.

[51] Philbrick JT, Becker DM (1988). Calf deep venous thrombosis. A wolf in sheep’s clothing?. Arch Intern Med.

[52] Prandoni P, Lensing AW, Prins MH (2004). Below-knee elastic compression stockings to prevent the post-thrombotic syndrome: a randomized, controlled trial. Ann Intern Med.

[53] Cohen JM, Akl EA, Kahn SR (2012). Pharmacologic and compression therapies for postthrombotic syndrome: a systematic review of randomized controlled trials. Chest.

[54] Kahn SR, Comerota AJ, Cushman M (2014). The postthrombotic syndrome: evidence-based prevention, diagnosis, and treatment strategies: a scientific statement from the American Heart Association. Circulation.

[55] Chaer RA, Myers J, Pirt D (2010). The impact of a systemwide policy for emergent off-hours venous duplex ultrasound studies. Ann Vasc Surg.

[56] Wells PS, Anderson DR, Bormanis J (1997). Value of assessment of pretest probability of deep-vein thrombosis in clinical management. Lancet.

[57] Raja AS, Greenberg JO, Qaseem A (2015). Evaluation of patients with suspected acute pulmonary embolism: best practice advice from the Clinical Guidelines Committee of the American College of Physicians. Ann Intern Med.

[58] Righini M, Van Es J, Den Exter PL (2014). Age-adjusted D-dimer cutoff levels to rule out pulmonary embolism: the ADJUST-PE study. JAMA.

[59] Goodacre S, Sampson FC, Sutton AJ (2005). Variation in the diagnostic performance of D-dimer for suspected deep vein thrombosis. QJM.

[60] Jennersjo CM, Fagerberg IH, Karlander SG (2005). Normal D-dimer concentration is a common finding in symptomatic outpatients with distal deep vein thrombosis. Blood Coagul Fibrinolysis.

[61] Wildberger JE, Vorwerk D, Kilbinger M (1998). Bedside testing (SimpliRED) in the diagnosis of deep vein thrombosis. Evaluation of 250 patients. Invest Radiol.

[62] Adhikari S, Zeger W, Thom C (2015). Isolated deep venous thrombosis: implications for 2-point compression ultrasonography of the lower extremity. Ann Emerg Med.

[63] Bramante RM, Raio CC (2013). Near-miss in focused lower-extremity ultrasound for deep venous thrombosis. J Emerg Med.

[64] Ramsey SC, Flaherty PM (2015). The unlikely presence of deep vein thrombosis in a patient with low pretest probability and a negative D-dimer: a case report. J Emerg Med.

[65] Galanaud JP, Sevestre MA, Genty C (2010). Comparison of the clinical history of symptomatic isolated muscular calf vein thrombosis versus deep calf vein thrombosis. J Vasc Surg.

[66] Sales CM, Haq F, Bustami R (2010). Management of isolated soleal and gastrocnemius vein thrombosis. J Vasc Surg.

[67] Schwarz T, Buschmann L, Beyer J (2010). Therapy of isolated calf muscle vein thrombosis: a randomized, controlled study. J Vasc Surg.

